# Offenheit in der IT: Herausforderung und Chance zu Veränderungen

**DOI:** 10.1365/s40702-021-00712-4

**Published:** 2021-03-06

**Authors:** Matthias Knoll

**Affiliations:** Hochschule Darmstadt, Haardtring 100, Darmstadt, Deutschland

**Keywords:** Offenheit, Freiheit, Open Definition, Mindset, Open Hardware, Open-Source-Software, FLOSS, Openness, Freedom, Open Definition, Mindset, Open Hardware, Open Source Software, FLOSS

## Abstract

Wer offene Lösungen einsetzen möchte, sieht sich einer Reihe von Fragen und Herausforderungen gegenüber. Nicht nur technische Aspekte, sondern vor allem zwischenmenschliche Faktoren und passende Regelungen sind dabei zu berücksichtigen. Das richtige „Mindset“ spielt eine zentrale Rolle. Mit ihm einher geht eine lebendige Umsetzung des Community-Gedankens. Er ermöglicht, die Idee der Offenheit über Open Source und Open Hardware hinaus auch auf Fragen des Identity Managements, der Entwicklung von Innovationen oder die Nutzung von Open Data auszudehnen. Hierzu bedarf es neben technischem Verständnis vor allem auch Akzeptanzkriterien und einem Bewusstsein für Risiken. Der Beitrag verfolgt daher das Ziel, einen Überblick über die Themen und Fragestellungen zu geben und eine Empfehlung für den Umgang mit Offenheit in der IT zu formulieren.

## Abstract (en)

Anyone who wants to deploy open solutions faces a number of questions and challenges. Not only technical aspects, but above all interpersonal factors and appropriate regulations must be taken into account. The right “mindset” plays a vital role. It goes hand in hand with a lively implementation of the community idea. It makes it possible to extend the idea of openness beyond open source and open hardware to questions of identity management, the development of innovations or the use of open data. This requires not only technical insights, but also acceptance criteria and risk awareness. This article therefore aims to provide an overview of key issues and to formulate a recommendation for dealing with openness in IT.

## Der Begriff der Offenheit

Offenheit ist ein komplexer Begriff. Um ihn zu strukturieren, hat die Open Knowledge Foundation eine allgemeine Definition erarbeitet, die aktuell in der Version 2.1 vorliegt (Open Knowledge Foundation 2015). In verkürzter Form definiert sie „offen“ als „anyone can freely access, use, modify, and share for any purpose (subject, at most, to requirements that preserve provenance and openness)“.

Damit diese Definition greifbarer wird, sind einige Bedingungen zu beachten, die im Kontext von Offenheit gelten müssen (Bauer et al. 2015):

- Digitale Bereitstellung aller Elemente für bestmögliche Sichtbarkeit, Nutzbarkeit, Teilbarkeit und Modifizierbarkeit
- Auffindbarkeit/Sichtbarkeit, denn nur dann können die Elemente auch tatsächlich genutzt werden
- Teilhabe im Sinne eines barriere- und diskriminierungsfreien Zugangs
- Nachvollziehbarkeit, um die Entstehungsgeschichte transparent zu machen und so Missverständnissen vorzubeugen

Da IT-Themen nicht ausschließlich technisch diskutiert werden sollen, erscheint es sinnvoll, den Begriff der Offenheit auf obiger Basis weiter zu präzisieren und dazu mehrdimensional zu betrachten. Drei Dimensionen bedingen sich dabei gegenseitig:

1. die menschliche/soziale Dimension
2. die technische Dimension
3. die prozessuale Dimension

Ein integrierender Blick auf alle drei Dimensionen erschließt die vielfältigen Chancen von Offenheit in der IT.

1. *Die menschliche/soziale Dimension*
  Gute IT-Lösungen leben von Interdisziplinarität. Gerade im Kontext der Digitalisierung ist die Zusammenarbeit zwischen den Spezialisten in den Fachabteilungen und in der IT wichtiger denn je, um notwendige Veränderungen erfolgreich umsetzen zu können. Hierfür müssen alle Beteiligten bestimmte Eigenschaften in die Kommunikation und Kooperation einbringen. Offenheit ist eine solche wesentliche Eigenschaft. Sie kann mit *Aufrichtigkeit, Aufgeschlossenheit* und der Bereitschaft erklärt werden, *sich mit Personen, Fragen und Problemen unvoreingenommen auseinanderzusetzen *(Duden 2020; Bedeutung Nr. 2). Einzelne Fachdisziplinen, wie etwa die Psychologie, ergänzen diese Definition mit Blick auf Persönlichkeitseigenschaften und Verhaltensweisen. Dazu zählen neben einer Dialogkultur Einfallsreichtum und Erfindergeist, Originalität, Fantasie, intellektuelle Neugierde und Interesse an Ästhetik sowie Ehrlichkeit, Optimismus, Toleranz, Vorlieben für Abwechslung und neuen Aktivitäten (Tretter 2016; McCrae 2007). Häufig schätzen sich Menschen als offen ein, wenn sie aufmerksam für eigene und fremde Emotionen sind. Offenheit lässt sich also mit einer bestimmten Geisteshaltung und inneren Einstellung (im Sinne von Persönlichkeitseigenschaften) erklären und setzt letztlich entsprechende Denk- und Verhaltensmuster (das *Mindset*) voraus (Hawlitzeck 2018; Hruby und Hanke 2014). Es ist also wahrscheinlich und für das Thema Offenheit ein besonders zentraler Faktor, dass sich Menschen mit ähnlichen Einstellungen und Werten in Bezug auf Offenheit gerne einer Gruppe Gleichgesinnter anschließen. Denn nur in einer solchen Gruppe kann dem Gedanken der Offenheit Gehör verschafft und können darauf basierende Lösungen etabliert werden. Je größer und einflussreicher diese Gruppe ist und je größer die Gemeinsamkeiten sind, desto höher ist die Wahrscheinlichkeit, dass sich Offenheit in den jeweiligen Bereichen der IT, in denen diese Gruppen aktiv sind, etablieren kann. Ein Beispiel für eine solche Entwicklung ist die Entstehungsgeschichte von Linux (Moody 2001). Dabei ist es unerheblich, ob sich die Gruppenmitglieder ausschließlich durch Kontakte in der virtuellen Welt kennen oder auch durch Kontakte in der realen Welt oder einer Kombination aus beidem.
2. *Die technische Dimension*
  Alle technischen Lösungen lassen sich auf einem Kontinuum zwischen *vollständig geschlossen* und *vollständig offen* einordnen. Nicht nur in der Wirtschaftsinformatik gilt Offenheit vielfach als wichtiger Treiber für innovative Lösungen (Open Innovation, Chesbrough 2003, 2019). Technische Lösungen, deren Spezifikationen und Funktionsweisen bewusst und gewollt offengelegt werden und damit Gegenstand von fachlichen Diskursen und betriebswirtschaftlichen Planungen sind, gewinnen an Reife und beantworten bislang bestehende Fragen in Forschung und Anwendung. Ein Beispiel aus der IT-Geschichte ist der IBM PC, dessen Architektur durch IBM selbst von Beginn an und gewollt offengelegt wurde (Borchers 2011). Umgekehrt können auch innovative IT-Ansätze zu mehr Offenheit führen (Schlagwein et al. 2017).
  Ein weiteres zentrales Merkmal offener Systeme ist ihre wechselseitige Kompatibilität. Die Kompatibilität kann sich dabei auf den Datenaustausch beschränken, oder aber durch Wahl geeigneter Technologien zu weitgehender Unabhängigkeit von Hardware- und Betriebssystemlösungen oder von einer bestimmten Anwendungssoftware führen. Eine zu beachtende Randbedingung ist dabei, dass mit zunehmender Offenheit eines Systems die Gefahr der Fragmentierung zunimmt (Parker et al. 2016), wie etwa an der Vielzahl von Linux-Distributionen und an Abspaltungen (Forks) bekannter Lösungen deutlich wird. Durch sie kann der zentrale Vorteil der Kompatibilität deutlich verringert oder im Extremfall sogar aufgehoben werden.
3. *Die prozessuale Dimension*
  Damit sowohl in der 1. als auch in der 2. Dimension Offenheit nachhaltig möglich ist, müssen grundsätzliche Regelungen für die damit verbundenen Prozesse und Aktivitäten und die darin genutzten Ressourcen getroffen werden. Dies gilt überall dort, wo partizipativ gearbeitet wird und demokratische Grundsätze angewandt werden. Damit verbunden ist stets der Wille zu Transparenz und zum Aufbau organisatorischer und technischer Netzwerkstrukturen als zentrale Unterstützungselemente. Regelungen können dabei formell oder informell vereinbart werden. Zentrale Bedingung ist, dass diese Regelungen allgemein akzeptiert sind.
  Kooperationsprozesse und damit die Ausgestaltung der Zusammenarbeit (beispielsweise alle Formen von Abstimmungen, Entscheidungsbefugnisse sowie Rollen/Gremien) sollen klar geregelt werden, ebenso Prozesse für den freien und offenen Zugang zu Informationen und die ungehinderte Kommunikation (Buchner und Schmelzer 2003).

## Teilbereiche der Offenheit

**Abb. 1:**
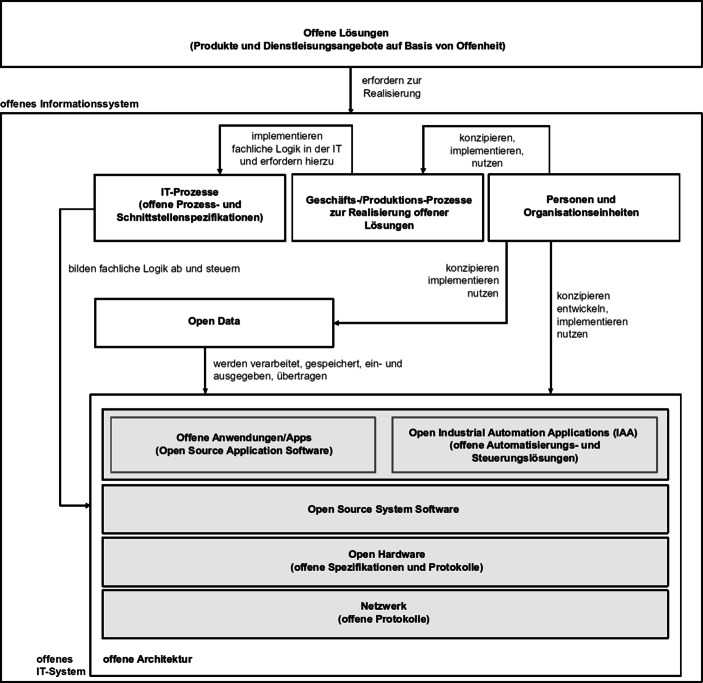
Offenes Informationssystem

Im Kontext der Nutzung von IT hat sich für eine Strukturierung das Konzept des Informationssystems etabliert (Abts und Mülder 2017). Dieser Ansatz beschreibt ein soziotechnisches System, das aus verschiedenen, jeweils voneinander unabhängigen Teilen besteht, die jedoch miteinander interagieren. Sie dienen einer Organisation stets zur Unterstützung oder Realisierung fachlicher Fragestellungen. Entsprechend ermöglichen offene Informationssysteme (vgl. Abb. 1) offene Lösungen.

Offenheit schließt dabei alle Elemente des Informationssystems in unterschiedlicher Form und Ausprägung ein.

### Personen und Organisationseinheiten

Unter Organisationen werden alle staatlichen Einrichtungen und branchenübergreifend alle privaten Unternehmen sowie alle darin beschäftigten Personen subsummiert.

Um Offenheit erfolgreich implementieren zu können, müssen Organisationen mit Blick auf die Dimensionen 1 und 3 aus Abschn. 1 eine Kultur der Aufgeschlossenheit zum Thema Offenheit pflegen und entsprechende Richtlinien vorgeben, die es ihren Mitgliedern ermöglicht, entsprechend den in der Open Definition niedergelegten Bedingungen zu handeln. Die Mitglieder der Organisation wiederum müssen über ein Mindset verfügen, das es erlaubt, Informationen und darauf basierendes Wissen ohne Angst vor Machtverlust oder Verlust an fachlichem Einfluss zu teilen.

Für Organisationen hat Offenheit den Vorteil, dass immer komplexere Sachverhalte gemeinsam betrachtet und im Idealfall besser und schneller Lösungen gefunden werden können. Hierzu binden emanzipatorische Modelle Know-how aus unterschiedlichen fachlichen und gesellschaftlichen Bereichen, aber auch Altersstufen ein. Zudem begünstigt die Möglichkeit Dritter, an Projekten aktiv teilzuhaben die Motivation und Fähigkeiten derjenigen, die organisationsintern in das Projekt eingebunden sind (Bauer et al. 2015).

Mit Blick auf die 2. Dimension stehen Organisationen beispielsweise vor der Frage, ob zur Feststellung der Identität des eigenen Personals, aber auch der externen Partner, etwa Kunden und Lieferanten, Open-Identity-Lösungen eingesetzt werden sollen. Zwar nutzen zahlreiche Organisationen intern Single-Sign-On-Lösungen, die das Problem der hohen Anzahl von Nutzerkonten umgehen, doch sind diese Lösungen in der Regel proprietär. Hinzu kommt, dass durch neue digitale Geschäftsmodelle auch Zugänge für Kunden (und Lieferanten) bereitgestellt werden müssen. Geht man von einer künftig noch stärker zunehmenden Vernetzung der Partner aus, wird die Anzahl unterschiedlicher Nutzerkonten sowohl im kommerziellen als auch im staatlichen Kontext zunehmen. Denn bestimmte Gruppen in der Organisation benötigen bei anderen Organisationen Zugänge, um etwa statistische Meldungen an Behörden zu übermitteln oder beim Lieferanten bestellen zu können. Gleichzeitig müssen sich Privatpersonen vielfach neu registrieren, wollen sie die neuartigen, digitalen Angebote einer Organisation annehmen. Dass die Diskussionen um Open Identity und Trust Services an Bedeutung gewinnen, zeigen zahlreiche Forschungsaktivitäten wie in (Ehrlich et al. 2021) systematisiert und die jährlich stattfindenden Open Identity Summits.

### Geschäfts- und Produktionsprozesse

Neben betriebswirtschaftlich geprägten Geschäftsprozessen rücken im Kontext der Digitalisierung auch Produktions- und Steuerungsprozesse immer stärker in den Vordergrund. Denn neue Geschäftsmodelle, „intelligente“ Maschinen und zunehmende Vernetzung ermöglichen eine höhere Effektivität und Effizienz in der Produktion und der Anlagensteuerung („Industrie 4.0“). Gleichzeitig erfordert dies eine immer engere Verbindung mit betriebswirtschaftlichen Prozessen. Parallel dazu nimmt die Digitalisierung auch in der öffentlichen Verwaltung zu (eGovernment).

Um neue Geschäftsmodelle im Kontext von Offenheit erfolgreich entwickeln und implementieren zu können, greifen viele Organisationen in der 1. Dimension auf die Bildung von Communities zurück. Sie sollen so angelegt sein, dass der Zugang frei ist, dass also neue Partner zur Nutzung bestimmter Kernkompetenzen unkompliziert eingebunden werden können. Dabei unterstützen sich die Partner wechselseitig mit Informationen und Wissen, ohne sich Kompetenzen und Märkte streitig zu machen. Im Idealfall ist die Kompetenz in einem solchen Netzwerk so verteilt, dass immer ein Partner für ein spezielles Problem eine passende Lösung bereitstellen kann. Beispiele hierfür sind große Softwarehersteller, die neben kommerziellen auch Community-Versionen ihrer Lösungen anbieten und dafür externe, in der Regel ehrenamtlich tätige Spezialisten im Sinne des Open-Innovation-Gedankens aktiv in Entwicklung und Support einbinden. Mit Blick auf den Support gehen beispielsweise große Telekommunikationsunternehmen ähnlich vor. In deren Community helfen sich Kunden gegenseitig. Nur bei Bedarf unterstützen Experten aus der Organisation. Ob sich ein solcher Community-Ansatz im Bereich der Produktion, etwa mit Open Manufacturing (Bauwens 2009; Redlich und Bruhns 2008), ebenfalls durchsetzen kann, ist offen (Waldman Brown 2016).

In diesem Kontext wichtige Elemente der 2. Dimension sind offene Plattformen. Eine große Herausforderung etwa in Produktions- und Steuerungsprozessen ist die Verknüpfung heterogener Maschinen und Anlagen für eine optimale Steuerung und Kontrolle. Eine offene Lösung hat hier den Vorteil, dass eine Einigung darauf alle Beteiligten in gleicher Weise prozessual einbindet. Kein Hersteller oder Anbieter würde so bevorzugt. Konfliktbehaftete Fragen wie etwa die nach der Dateneigentümerschaft oder der Steuerungshoheit, ließen sich ergebnisorientierter diskutieren.

Um Prozesse beschreiben zu können, werden mit Blick auf die 3. Dimension geeignete Modellierungssprachen eingesetzt. Als de-facto-Standard gilt mittlerweile die zwar nicht offene, jedoch frei nutzbare Prozessbeschreibungssprache BPMN 2.0.2 der OMG (OMG 2013). Sie kann mit entsprechenden Erweiterungen, etwa der Industry 4.0 Process Modeling Language (I4PML, Petrasch und Hentschke 2016) oder der BPMN for Cyber Physical Systems (BPMN4CPS, Graja et al. 2016) auch im Produktionskontext für die fachliche Beschreibung der Prozesse eingesetzt werden.

### IT-Prozesse

IT-Prozesse implementieren die entwickelten Geschäfts‑, Produktions- und Steuerungsprozesse im IT-System. Ihr Ziel ist es, im IT-System laufende Prozesse zu steuern und dazu den Daten- und Kontrollfluss sicherzustellen.

Aus technischer Sicht, in der 2. Dimension, bedarf es hierzu offener Protokolle und Schnittstellen.

In der 3. Dimension sind einerseits zur technischen Modellierung der Prozesse, andererseits zur Implementierung von offenen Softwaresystemen (siehe Abschn. 2.5) Beschreibungssprachen hilfreich. Neben der BPMN für Geschäfts- und Verwaltungsprozesse, die auch technische Ressourcen abbilden kann, lassen sich I4PML, BPMN4CPS, aber auch die auf UML basierende, ebenfalls nicht offene, aber frei nutzbare SysML 1.6 der OMG (OMG 2019) für Produktions- und Anlagensteuerungsprozesse nutzen.

### Open Data

Daten gelten im Kontext der Digitalisierung als 4. Produktionsfaktor. Ihre systematische Gewinnung, Aufbereitung und Nutzung ist für die Gestaltung und den Erfolg neuer Geschäftsmodelle wesentlich, weshalb eine Veröffentlichung als Open Data nicht selbstverständlich ist, was jedoch mangelnde Transparenz zur Folge hat.

Daten sind mit Blick auf die 3. Dimension offen, wenn sie folgende Eigenschaften und Anforderungen erfüllen (Bauer et al. 2015; OKFN 2020). Sie

- sind Primärdaten
- werden unter freien Lizenzen für jegliche (auch kommerzielle) Anwendung bereitgestellt (Reuse & Redistribution)
- sie werden unter offenen Standards publiziert
- sind dauerhaft verfügbar (Availability & Access)
- sind maschinenlesbar
- haben keinen Personenbezug
- sind ohne infrastrukturkritischen Inhalte
- erlauben universelle, diskriminierungsfrei Partizipation (Universal Participation)

Open Data können im Sinne der 1. Dimension durch öffentliche Bereitstellung an Qualität und Detailliertheit gewinnen, wenn sie durch eine Community gepflegt werden, die von der Sache überzeugt ist. Freie Inhalte sind auf diesem Weg mittlerweile zu einem wichtigen Bestandteil des Alltags geworden. Als eines der bekanntesten Beispiele für themenübergreifend freie Inhalte gilt die freie Enzyklopädie Wikipedia, die in verschiedenen Sprachen durch eine große Community gepflegt wird. Andere freie Daten fokussieren auf ein bestimmtes Anwendungsgebiet, etwa frei verfügbare Geodaten im ebenfalls Community-unterstützten OpenStreetMap. Können Daten nicht vollständig offengelegt werden, kann auch eine Bereitstellung innerhalb einer größeren, jedoch geschlossenen Gruppe bereits ein großer Fortschritt sein. Ein wichtiges Beispiel hierfür ist der branchen- und organisationsübergreifende Datenaustausch im Kontext von Cyber-Angriffen mit dem Ziel einer verbesserten, gemeinsamen Abwehrstrategie (Hinz 2017).

Aus technischer Sicht (2. Dimension) mit der Bereitstellung von Open Data eng verbunden ist auch die Frage nach offenen Datenformaten. Sie beschreiben, wie detailliert Sachverhalte beschrieben werden und wie zwei Systeme miteinander Daten austauschen können. Sie entscheiden damit maßgeblich über die Leistungsfähigkeit von Datenbeständen. Die Wahl für ein bestimmtes Datenformat sollte daher sorgfältig getroffen werden. Typische offene Datenformate sind beispielsweise CSV und XML, aber auch Datenformate für Office-Dokumente (OpenDoument-Format ODF), Bild- (PNG) und Audiodateien (FLAC) oder Videodateien (Theora).

### Offene Systeme

In der Informationstechnik ist ein „offenes System“ eine Systemumgebung, die Interoperabilität, Portabilität und Erweiterbarkeit durch offene Schnittstellen und Spezifikationen sichert. Im Vordergrund stehen dabei die Unabhängigkeit von bestimmten Technologien, technischen Plattformen (Architekturen) und Herstellern (Hühnlein 2014). Der Anteil offener, transparenter und kooperativer Ansätze kann dabei – auch abhängig von der betrachteten Ebene – variieren. Er reicht von offenen Netzwerkprotokollen über die Offenlegung von Hardwarespezifikationen, über offene Systemsoftware bis zum komplexen quelloffenen Anwendungs‑, Automatisierungs- und Steuerungssystem (vgl. Abb. 1). Von besonderem Interesse für die weitere Diskussion sind dabei Open Source Software und Open Source Hardware.

#### Open Source Software

Software gilt dann als Open Source bzw. frei, wenn die in der Open Source Definition der Open Source Initiative (OSI 2017; Fox 2006) niedergelegten Forderungen erfüllt sind:

- die Möglichkeit zur freien Weiterverteilung
- den Zugriff auf den Quellcode
- die Möglichkeit zur Modifikation
- die Sicherstellung der Integrität des von Autoren verantworteten Quellcodes
- keine Diskriminierung gegenüber Personen und Gruppen
- die unveränderte Weitergabe der Lizenz
- keine produktspezifischen Lizenzen
- keine Einschränkungen anderer Lösungen durch die verwendete Lizenz
- Technologieneutralität

Auch die Free Software Foundation sieht das ähnlich, wobei die Diskussion über die „richtige“ Bezeichnung (Open Source versus Free Software) bis heute andauert (Ferraro und Torrents 2016; Jaeger und Metzger 2020). Um Uneinigkeit zu vermeiden, wird Software, die diese Bedingungen erfüllt, übergreifend als FOSS bzw. FLOSS bezeichnet. FOSS ist ein Akronym für „Free and Open Source Software“, weil jedoch „frei“ insbesondere nach Vorstellung der Free Software Foundation nicht mit Kostenfreiheit gleichgesetzt werden soll und darf, hat sich der Begriff FLOSS etabliert. Das „L“, aus „libre“ (französisch und spanisch), „livre“ (portugiesisch) oder „libero“ (italienisch) beschreibt darin den Freiheitsaspekt eindeutiger als das deutsche „frei“ oder das englische „free“.

Open Source Software findet sich heute in einer Vielzahl von Systemsoftware (einschließlich Infrastruktur-Komponenten zur Containerisierung, Orchestrierung, Provisionierung und zum Konfigurationsmanagement) sowie von Anwendungen, seien es betriebswirtschaftlich genutzte Anwendungssysteme oder Anwendungen im technischen Umfeld. Open Source Software kann zudem in Komponenten enthalten sein, bei denen die Software (Firmware) eng mit der Hardware verbunden ist. Netzwerkgeräte wie Router und Firewalls oder Switches, aber auch IoT-Geräte wie beispielsweise Maschinen und Anlagen mit ihren Sensoren und Aktoren, Messgeräte sowie viele Mobilgeräte gehören dazu. Und schließlich wird Open Source Software im Rahmen der Software-Entwicklung umfassend eingesetzt (Thelen et al. 2020).

Um solche Lösungen anbieten zu können, kooperieren vormals Hersteller von Closed-Source-Lösungen, aber auch Neugründungen zunehmend mit Open-Source-Entwicklern. Im Wesentlichen lassen sich dafür 3 Gründe nennen (Ferraro und Torrents 2016):

1. Die Möglichkeit für Innovationen abseits strikter Patentschutzaspekte
2. Die Möglichkeit der Bindung von Experten auf Basis von Freiwilligkeit und Schaffung einer nachhaltigen Community, ohne dabei bestehende Unternehmenszwänge beachten zu müssen
3. Die Möglichkeit zum Aufbau alternativer Geschäftsmodelle, nicht zuletzt auch mit dem Ziel, verschiedene Alternativen zu evaluieren

#### Open Hardware

Die konsequente Fortführung des Gedankens quelloffener Software auch im Hardware-Kontext besteht in der Offenlegung aller Informationen im Rahmen der Entwicklung (Open Source Hardware, OSHW). Die Hardwareentwicklung folgt dabei bestimmten Grundsätzen:

- Alle Informationen zum Schaltungsdesign und ggf. speziellen Hinweisen zur Herstellung werden veröffentlicht.
- Jeder Interessierte ist auf Basis dieser Informationen in der Lage, die Hardware selbst herzustellen oder unter Aufsicht herstellen zu lassen.
- Das fertige Bauteil muss erstmals vollständig und später aus der laufenden Produktion stichprobenartig strengen Tests unterzogen werden, gleiches gilt für die Firmware und alle weiteren Software-Elemente, die zum Betrieb unmittelbar notwendig sind. Ihr Quellcode muss offengelegt werden.

Einschlägige Projekte werden wegen des häufig hohen Finanzbedarfs in der Regel über große Crowdfunding-Plattformen initiiert.

## Akzeptanzkriterien

Für den erfolgreichen Einsatz von offenen Lösungen müssen verschiedene, teilweise wechselseitig voneinander abhängige Akzeptanzkriterien erfüllt sein. Sie lassen sich in vier große Bereiche unterteilen:

1. Erfüllung von Qualitätsmerkmalen
2. Einsatz von Standards und Best Practices
3. Beachtung von Compliance und Schaffung von Rechtssicherheit
4. Erfüllung weiterer Rahmenbedingungen

### Erfüllung von Qualitätsmerkmalen

Die ISO/IEC-Normenreihe 9126 beschreibt ein Modell für wichtige Software-Qualitätsmerkmale, deren Grundgedanken sich jedoch auch auf Hardware und Prozesse sowie Daten und Services und damit auch auf offene Lösungen insgesamt übertragen lassen.

Unter dem Faktor Funktionalität stellt der Faktor Sicherheit für die Akzeptanz offener Lösungen ein zentrales Kriterium dar. Entsprechend kontrovers wird diskutiert. Denn die Offenlegung von Schaltungsdesign und Programmcode kann einerseits für ein umfassendes Review, andererseits aber auch für die intensive Suche nach Schwachstellen genutzt werden. Unstrittig ist, dass erkannte Schwachstellen rasch geschlossen werden könnten, allerdings zeigen viele Fälle, wie etwa der bekannte Heartbleed-Bug im OpenSSL-Code (Synopsis Inc. 2020), aber auch aktuelle Studien, dass Schwachstellen oft jahrelang unentdeckt bleiben (GitHub 2020). Je besser es also einem Projekt gelingt, Schwachstellen durch geeignetes Design zu vermeiden oder zumindest je schneller es gelingt, sie in der eigenen Lösung zu identifizieren und zu beseitigen, desto höher wird die Akzeptanz sein. Sicherlich auch aus diesem Grund hat sich 2020 die Open Source Security Foundation als Initiative der Linux Foundation gegründet (Hahn 2020). Sie soll unternehmens- und branchenübergreifend die Sicherheit von Open Source Software verbessern. In ihr geht die bereits bestehende Initiative Open Source Security Coalition auf, auch die Core Infrastructure Initiative wird sich vermutlich anschließen. Ziel laut Ankündigung im Blog der Linux Foundation ist das Entwickeln von Best Practices der IT-Sicherheit für quelloffene Software (Hahn 2020). Bei diesem überaus kritischen Thema wäre eine Beteiligung aller wesentlichen kommerziellen und nicht-kommerziellen Stakeholder von großer Bedeutung. Positiv ist dabei, dass Best Practices keine strikten Vorgaben machen, sondern sich auf die individuellen Verhältnisse anpassen lassen und dadurch die Akzeptanz stärken.

Weitere wichtige Faktoren für Qualität sind Zuverlässigkeit, Benutzbarkeit, Effizienz, Änderbarkeit und Übertragbarkeit. So können beispielsweise hochgradig verteilte und virtualisierte Entwicklungsprozesse im Open-Source-Umfeld die Zuverlässigkeit der Lösung beeinträchtigen. Um dem entgegenzuwirken, übernehmen vielfach Unternehmen oder Stiftungen die Führungsrolle und sorgen für eine geordnete Entwicklungsarbeit.

Für Unternehmen, die bereits offene Lösungen einsetzen oder überlegen, dies zu tun, könnte also eine Empfehlung sein, sich – schrittweise – aktiv in nationale und internationale Gremien und Communities einzubringen. Dies könnte zunächst etwa dadurch realisiert werden, dass einzelne Experten Freiräume für Aktivitäten beispielsweise auch in Foren und Entwicklungsprojekten erhalten, was auch Teil einer Incentivierung sein könnte. Später dann kann eine offizielle Mitwirkung des Gesamtunternehmens, auch finanziell, vorgesehen werden, etwa auch in Stiftungen oder durch Übernahme der Entwicklungskoordination.

### Einsatz von Standards und Best Practices

Für den Erfolg bei Entwicklung und Einsatz offener Lösungen haben offene Standards eine hohe Bedeutung (Hühnlein 2014). Denn etablierte Standards sichern insbesondere Interoperabilität und erleichtern den Austausch ungeeignet erscheinender Elemente. Dass Standardisierung jedoch nicht grundsätzlich notwendig oder sinnvoll sein muss, insbesondere, wenn sie sehr weitreichend sein will, zeigt die kritische Diskussion um die Vielzahl an Linux-Distributionen, die vielfach unterschiedliche Konzepte, etwa hinsichtlich der Struktur des Filesystems und wichtiger Systembibliotheken, vor allem aber der Software-Paketierung (beispielsweise rpm, apt, yum) einsetzen. Um zentrale Aspekte zu standardisieren und so nicht zuletzt die Distributions-übergreifende Anwendungsentwicklung und den Support zu vereinfachen, hat die Linux Foundation die Initiative Linux Standard Base (Linux Foundation 2017) ins Leben gerufen, die auch die Arbeiten an einer einheitlichen Filesystem-Systematik umfasst („Filesystem Hierarchy Standard“, FHS). Allerdings sind aktuell nur sehr wenige Distributionen vollständig LSB-konform. Viele große Distributionen lehnen wegen des damit verbundenen hohen Zertifizierungsaufwandes eine umfassende LSB-Zertifizierung ab. Lediglich die Struktur des Filesystems sowie einige wenige zentrale Boot-Routinen (beispielsweise der Init-Prozess) folgen dem Standard. Das ist einerseits aus konzeptioneller Sicht bedauerlich, weil es eine umfassende Cross-Plattform-Nutzung von Anwendungen sowie Zertifizierungsbemühungen erschwert und zur Zersplitterung des Distributions-Spektrums beiträgt. Andererseits ist ein geringer Grad an Standardisierung für die jeweiligen Distributionen aus ökonomischer Sicht sowie aus Autonomiegesichtspunkten verständlich. So bleibt – besonders zentral im Kontext der Offenheit – der Anwendungsseite weiterhin die Wahlfreiheit, es können Einsatzschwerpunkte definiert, Innovationen gewagt und eine Machtkonzentration vermieden werden (Powers 2008).

Für den Einsatz in Unternehmen kann dies jedoch tatsächlich ein Problem sein. Hilfreich könnte daher bei der Wahl beispielsweise einer Linux-Distribution sein, sich an Marktanteilen und begleitenden kommerziellen Support-Angeboten zu orientieren oder gezielt durch Einstellung von Spezialisten eigenes Know-how aufzubauen und sich dadurch unabhängig zu machen.

Im Kontext der Entwicklung offener Hardware leistet die Open Source Hardware Association (oshwa.org) einen wichtigen Beitrag zur Akzeptanz. Denn Ziel dieser Organisation ist es, bei der Vermittlung von Wissen über aktuelle Technologien zu unterstützen und entsprechende didaktische Konzepte aktiv zu fördern. Gleichzeitig soll Forschung, deren Ergebnisse für alle zugänglich sind, an der alle mitwirken können und die die Freiheitsrechte der späteren Nutzer respektiert, ebenfalls aktiv gefördert werden.

Um dieses eher allgemein gehaltene Ziel erreichen zu können, hat die OSHWA gut auf die jeweiligen Projekte und Anwendungsfälle anpassbare Best Practices auch in deutscher Sprache formuliert, die dabei helfen sollen, quelloffene Hardware zu ermöglichen (OSHWA 2013).

Parallel zu diesen Bemühungen existiert seit Sommer 2020 ein Vorschlag für eine Norm im Kontext der Entwicklung offener Hardware, die DIN SPEC 3105 (Meyer 2020). Der Vorschlag erscheint – konsequenterweise – unter der Creative Commons BY-SA 4.0 und kann im Unterschied zu anderen Normen kostenlos bezogen werden.

Um die Entwicklung offener Hardware möglichst umfassend zu unterstützen, ist der Normvorschlag in zwei Teile gegliedert:

- „Teil 1: Anforderungen an die technische Dokumentation“ (DIN SPEC 3105-1:2020-07) definiert grundlegende Begriffe und klare Anforderungen an die technische Dokumentation offener Hardware.
- „Teil 2: Community-basierte Bewertung“ (DIN SPEC 3105-2:2020-07) definiert ein in der Praxis umsetzbares Verfahren, mit dem Open-Source-Hardware-Produkte transparent und dezentral bewertet werden können – ähnlich den Peer-Reviews bei wissenschaftlichen Arbeiten.

Für die Praxis könnten Open-Source-Hardware-Entwicklungen eine Möglichkeit sein, Abhängigkeiten von Herstellern zu reduzieren oder die Interoperabilität zu verbessern. Auch die gefürchteten „Abkündigungen“ von Produkten oder deren zentrale Komponenten könnten damit besser beherrscht werden, weil offengelegte Spezifikationen einen Nachbau zumindest leichter ermöglichen.

Standards und Best Practices haben in Form von Zertifizierungen auch in der Aus- und Weiterbildung eine hohe Bedeutung, da sie eine gewisse Vergleichbarkeit ermöglichen. Gleichzeitig ist ihr Nutzen umstritten, denn mit ihnen wird zunächst nur das Beherrschen der Zertifizierungsinhalte, nicht jedoch eine umfassende Erfahrung nachgewiesen. Für Unternehmen könnten Zertifizierungen daher eine wichtige Orientierung bei der Auswahl des Personals sein, sollten jedoch übliche Personalrekrutierungsmaßnahmen lediglich flankieren.

Im Open-Source-Kontext existieren zahlreiche Zertifizierungsprogramme. So bieten etwa das Linux Professional Institute sowie die Linux Foundation als neutrale Organisationen Zertifizierungen zu unterschiedlichen Themen an (LPI 2020; LFT 2020). Auch größere Branchenverbände haben Linux-Zertifizierungen im Programm, ebenso Unternehmen wie RedHat und SUSE, die jedoch produktbezogen ausbilden (RedHat 2020; SUSE 2020).

### Beachtung von Compliance und Schaffung von Rechtssicherheit

Ein zentrales Akzeptanzkriterium ist zum einen die Schaffung von Rechtssicherheit. Dies betrifft den Umgang mit Lizenzen und Fragen des Urheberrechts, aber auch Gewährleistungs‑, Haftungs- und Supportgarantien, ehe Open-Source-Lösungen, insbesondere im kommerziellen Umfeld, genutzt werden können. Denn rechtliche Fragen im Kontext von offenen Lösungen sind komplex. Bereits die Vielzahl parallel existierender Open-Source-Lizenzen macht die Problematik deutlich. Als Grundtypus und weitverbreitetste Lizenz gilt die GNU General Public Licence, die aktuell in der Version 3 vorliegt (GPL v3.0; FSF 2020; Jaeger und Metzger 2020; Wilmer 2021).

Rechtssicherheit muss unabhängig davon gelten, ob Produkte für den Eigengebrauch entwickelt oder am Markt angeboten werden und ob die eingesetzte Open-Source-Software nur einen kleinen Teil der Funktionalität verantwortet oder für den Betrieb wesentlich ist. Zudem muss immer dann, wenn direkt oder indirekt unter Einbezug von Open-Source-Software kommerzielle Ziele verfolgt werden, sowohl auf Anbieter-, als auch auf Nutzerseite genau geprüft werden. Denn ohne Rechtssicherheit können Prozessrisiken entstehen. Auch fehlt die Möglichkeit zur Vereinbarung und Durchsetzung von Service-Leveln, die für den Einsatz bestimmter, insbesondere unternehmenskritischer Lösungen (etwa Speicherlösungen, Firewalls, Audio‑/Video-Kommunikationsplattformen) unabdingbar sind. Um mit Open-Source-Lösungen kommerziell erfolgreich sein zu können, beschreiten daher zahlreiche IT-Unternehmen den Weg, sich unmittelbar an der Entwicklung von Open-Source-Lösungen zu beteiligen und ihre Entwicklungen beispielsweise sowohl als offene Community Edition mit eingeschränkter Funktionalität, als auch als kostenpflichtige Enterprise Edition mit speziellen, im kommerziellen Kontext häufig geforderten (Closed-Source‑)Funktionalitäten und entsprechenden vertraglichen Garantien anzubieten.

Ein weiteres zentrales Akzeptanzkriterium ist zum anderen, ein bestehendes organisationsinternes Compliance-System (etwa nach dem Vorbild des Open-Chain-Projekts, OpenChain 2020; Jaeger und Metzger 2020) um Regelungen für alle über die dargestellten Punkte hinausgehenden Fragen zu ergänzen oder über den Aufbau eines solchen Systems grundsätzlich nachzudenken. Auch ist es in jedem Fall sinnvoll, eine fachkundige interne oder externe Stelle zu benennen, die bei allen Rechtsfragen unkompliziert hinzugezogen werden kann, auch von Entwicklungsteams oder dem Support.

Im Kontext der Bereitstellung von Daten als Open Data (dazu zählt auch Prozesswissen, etwa im Industrie‑4.0‑Umfeld) ist Rechtssicherheit und Risiko-Bewusstsein für damit verbundene Fragen in besonderer Weise wichtig. Denn wer Daten bereitstellt, macht sich in vielerlei Hinsicht angreifbar. Daten können fehlerhaft sein und damit ein negatives Image auslösen oder verstärken. Daten können Know-how enthalten, die Basis für neue Geschäftsmodelle sein und damit die Wettbewerbsposition schwächen, wenn sie Dritten zur Verfügung stehen. Daten können zudem besonders schützenswerte Elemente enthalten, etwa einen Personenbezug. Eine unüberlegte Bereitstellung kann dann rasch rechtliche Konsequenzen zur Folge haben. Um die Bedingungen für die Bereitstellung solcher Inhalte differenziert regeln zu können, werden sie in der Regel unter einer der Urheberrechtslizenzen der Creative Commons (Cesarini 2020; CC 2020) oder der Open Knowledge Foundation (OKFN 2020) gestellt. Es bedarf darüber hinaus einer auf die jeweilige Organisation angepassten Data Governance, wie sie etwa in (Gluchowski 2020) vorgeschlagen wird. So können Optionen mit direkten oder indirekten Auswirkungen auf den Geschäftserfolg deutlich verbessert abgeschätzt und Entscheidungen fundierter getroffen werden. Neben der geübten Praxis einer sorgfältigen Klassifikation von Daten sowie der Vergabe von Berechtigungen durch klar definierte und damit verantwortliche Dateneigentümer, ist es im Kontext guter Data Governance mit Blick auf eine bereits geschaffene oder im Aufbau befindliche Community von zentraler Bedeutung, auf Basis klarer Vereinbarungen mit deren Mitgliedern eine Vertrauensbasis für den Umgang mit Daten zu entwickeln. Wer sich durch ein wechselseitiges Geben und Nehmen sowie durch den schrittweisen Auf- und Ausbau eines Netzwerks darauf verlassen darf, dass „seine“ Community verantwortungsbewusst mit den bereitgestellten Daten umgeht, kann sie auch gewinnbringend einsetzen. So kann beispielsweise die Community herangezogen werden, um Fehler in den Daten zu identifizieren und zu beheben oder um darauf aufbauend neue Geschäftsmodelle oder andere interessante Nutzungsformen zu entwickeln, etwa auch zur Verbesserung interner Prozesse.

In einigen Fällen kann es notwendig und wichtig sein, Sachverhalte juristisch formal schriftlich zu fixieren. In anderen Fällen kann es erfolgversprechender sein, in der Sprache der Community geeignete Formulierungen zu wählen, ohne dabei das Ziel der Vereinbarung zu verwässern. Hierbei ist entsprechende juristische Beratung besonders wichtig.

### Erfüllung von Rahmenbedingungen

Eine Kostenbetrachtung ist für die Entscheidung „Pro oder Contra Offene Lösungen“ eine wichtige Rahmenbedingung. Kostenbetrachtungen sind stets fallbezogen und nur schwer zu verallgemeinern. Sie speisen sich unter anderem aus dem Erfüllungsgrad der drei vorausgehenden Akzeptanzkriterien und deren Merkmale bzw. Faktoren. Aber auch individuelle Erfahrungen im Umgang mit offenen Lösungen und die Unternehmensgröße sind von großer Bedeutung. Vielfach wird für eine möglichst differenzierte Bewertung der Kosten ein TCO-Modell genutzt (Shaik und Cornford 2011; O’Connell 2016). Hierin enthalten sind stets auch die Kosten für Support und Betrieb.

Offene Kommunikation innerhalb der Organisation ist eine zweite wichtige Rahmenbedingung. Denn nicht alle Mitglieder in der Organisation sind unmittelbar von der Idee der Offenheit überzeugt. Dies gilt insbesondere dann, wenn etwa Daten als Open Data bereitgestellt werden sollen. Eigeninteressen oder Ängste können dazu führen, dass der Versuch, ein offenes Mindset in der Organisation zu etablieren, bewusst oder unbewusst untergraben wird. In der Praxis muss es daher Ziel sein, Kritiker vom Nutzen der Offenheit für die eigene Position und die Organisation insgesamt immer wieder zu überzeugen. Neben Methoden wie etwa Newsletter, Workshops oder Gamification-Ansätze, aber auch E‑Learing (Europäische Kommission 2020) geschieht dies durch ein starkes Management-Commitment und nicht zuletzt durch persönliche Gespräche. Es soll klar kommuniziert werden, dass Veränderungen durch Offenheit auch die Leitungsebene(n) betreffen und dort akzeptiert, „gelebt“, ja sogar aktiv gefördert werden, um zukunfts- und wettbewerbsfähig bleiben zu können, und dass eine aktive Mitwirkung auf allen Ebenen durch eine geeignete Incentivierung honoriert wird.

Eine dritte wichtige Rahmenbedingung ist eine möglichst hohe Akzeptanz eines bestimmten Open-Source- oder Open-Hardware-Projektes in der Zielgruppe potenzieller Anwenderorganisationen. Ein Beispiel ist die Verwendung von offenen Entwicklungswerkzeugen oder Grafik- und Office-Tools. Fehlt diese Akzeptanz oder besteht eine Abhängigkeit in solchen Projekten von einer einzelnen Person, kann dies dazu führen, dass die Entwicklung eingestellt wird, wenn die Zielgruppe zu klein ist oder dieser Haupt-Unterstützer das Projekt verlässt – und im schlimmsten Fall weitere Team-Mitglieder mitnimmt. Daher muss vor dem Einsatz von bzw. Engagement für Open-Source-Lösungen genau untersucht werden, wie etabliert die Lösung ist und wie groß die Gruppe der aktiv Mitwirkenden ist. Eine umfassende Betrachtung mit Checkliste für diese Fragen, die auf weitere Lösungen übertragen werden kann, wird in (Thelen et al. 2020) vorgestellt.

Schließlich zählt auch die Frage der (Un‑)Abhängigkeit zu den Rahmenbedingungen. Auf Anwenderseite kann es von großer Bedeutung sein, wenn durch offenen Programmcode, offene Datenmodelle in Datenbanken sowie offene Schnittstellen ein Vendor-Lock-In minimiert wird und sich Systeme dadurch bedeutend einfacher migrieren lassen. Im Idealfall werden Entscheidungen für ein Open-Source-Tool immer auch nach dem Gesichtspunkt getroffen, dass durch Offenheit eine Migration in ein anderes Tool bei Bedarf einfacher ist, als ein Anbieterwechsel im proprietären Bereich, weil es am Markt ausreichend kommerzielle Angebote und Fachleute gibt, die solche Migrationen beherrschen, nicht nur einige wenige spezialisierte Anbieter.

## Ausblick und Versuch einer Empfehlung

Wer bereits positive Erfahrungen mit dem Thema Offenheit und offenen Lösungen gesammelt hat, wird eher dazu tendieren, den Gedanken der Offenheit weiterzutragen und entsprechende Vorgehensweisen und Lösungen zu empfehlen. Und tatsächlich lassen sich prinzipiell fast alle Fragestellungen mit offener Hard- oder Software oder frei verfügbaren Daten lösen. Doch nicht alles ist tatsächlich möglich, uneingeschränkt umsetzbar oder empfehlenswert. Denn vielfach treffen Nutzer offener Lösungen auf geschlossene Umgebungen, die – sieht man von nicht-zivilen Einsatzbereichen mit ihren besonderen Anforderungen ab – teilweise aus Bequemlichkeit, teilweise aus reinen Wirtschaftlichkeits- oder Risikogesichtspunkten, teilweise aber auch aus Unkenntnis oder diffuser Ablehnung heraus entstanden sind. Im Zeitablauf führen solche geschlossenen Umgebungen häufig zu hoher Akzeptanz und Bindung der Zielgruppe an das jeweilige Produkt oder die jeweilige Dienstleistung. Vielfach geschieht dies auch, indem die Lösung selbst kostenfrei – aber keinesfalls kostenlos oder gar frei – zur Nutzung überlassen wird.

Die Digitalisierung bietet jedoch sowohl auf Anbieter- als auch auf Anwenderseite zahlreiche Möglichkeiten für eine Öffnung, ohne dass auf Gewinne oder Marktanteile verzichtet werden muss. Vielmehr kann Offenheit gerade im Digitalisierungskontext auch als Zeichen von Innovation gesehen werden und dabei helfen, neue Kundengruppen zu erschließen. Auf beiden Seiten ist der Wille zur Offenheit eine Frage der organisationsspezifischen „politischen“ Position zu diesem Aspekt und eine Frage des Risikoappetits.

Es wird weder im Kontext staatlicher und privater Organisationen noch im privaten Umfeld möglich und sinnvoll sein, geschlossene Systeme und Lösungen vollständig zu substituieren. Das ist aber auch gar nicht notwendig und hilfreich, denn von geschlossenen Systemen können Impulse ausgehen, die auf offene Lösungen positiv Einfluss nehmen. Damit setzen sie vielleicht erst weitere Entwicklungen und Überlegungen in Gang, die zu einer Verbesserung und damit zu einer höheren Reife von IT-Lösungen insgesamt und einer verbesserten Interoperabilität und letztlich vielleicht zu einer Öffnung bisher geschlossener Lösungen beitragen.

Daher soll der Versuch einer Handlungsempfehlung darin bestehen, zu motivieren, den Einsatz offener und freier Lösungen sowohl im kommerziellen Umfeld als auch im Privaten unvoreingenommen zu erwägen und die sich bietenden Alternativen zu evaluieren.

Auch wenn es zu Beginn ein wenig mühsam sein kann, könnte mehr Offenheit auch unter Beachtung der Hinweise im vorausgegangenen Abschn. 3 schlussendlich zu mehr Vielfalt und Nachhaltigkeit führen (Bits und Bäume 2020). Dies gilt nicht zuletzt auch vor dem Hintergrund der COVID-19-Pandemie, in der rascher und ungehinderter Informationsaustausch und Open Access wichtige Elemente sind.

## Ausgewählte Internetquellen zum Thema Offenheit

- Creative Commons: https://creativecommons.org
- Free Software Foundation: https://www.fsf.org
- Linux Professional Institute: https://www.lpi.org/
- Linux Foundation: https://www.linuxfoundation.org
- Open Data Commons: https://opendatacommons.org
- Open Knowledge Foundation: https://okfn.org
- Open Source Hardware Association: https://www.oshwa.org
- Open Software Initiative: https://opensource.org
- Open Source Security Foundation: https://openssf.org
